# Supplementary material for: Diverse and Widespread Contamination Evident in the Unmapped Depths of High Throughput Sequencing Data
Source: PLoS One. 2014 Oct 29;9(10):e110808. doi: 10.1371/journal.pone.0110808 (PMC4213012; doi:10.1371/journal.pone.0110808)

Figure S1. Information per position in the RNAseq dataset. The entropy of each position in each of 460 libraries was calculated, and the distribution of these among datasets is depicted for each position. Due to the protocol used to generate these data, each read began with a six base pair barcode, followed by at least three guanines. On the basis of this analysis, the first fifteen bases, highlighted in gray, were trimmed from each read.

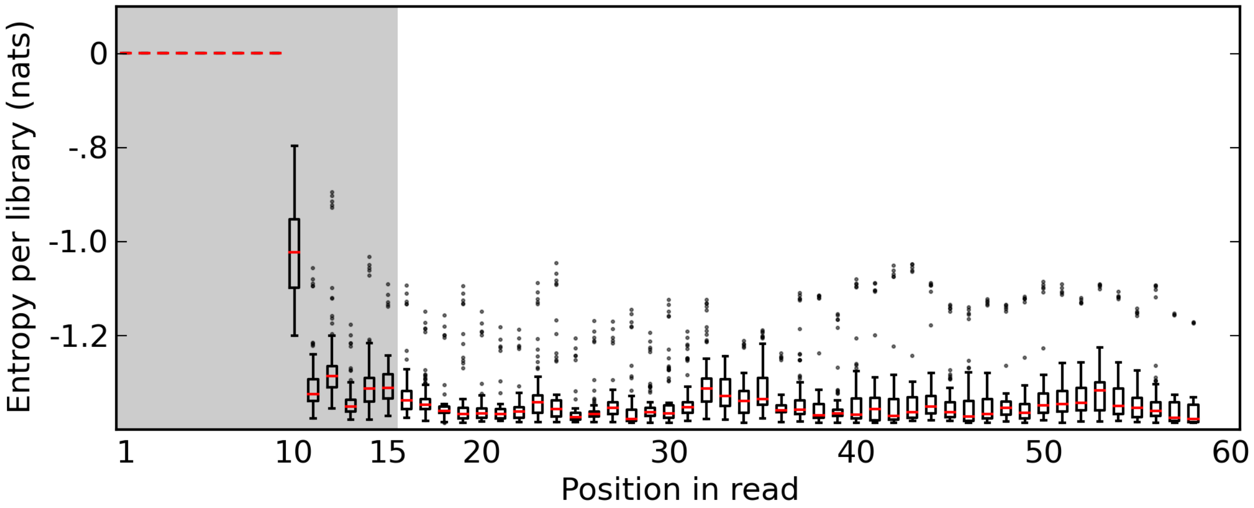

Supplement: Figure S1 — Information per position in RNAseq dataset. The entropy of each position in each of 460 libraries was calculated, and the distribution of these among datasets is depicted for each position. Due to the protocol used to generate these data, each read began with a six base pair barcode, followed by at least three guanines. On the basis of this analysis, the first fifteen bases, highlighted in gray, were trimmed from each read. (PDF) [file pone.0110808.s001.pdf]
