# Supplementary material for: Diverse and Widespread Contamination Evident in the Unmapped Depths of High Throughput Sequencing Data
Source: PLoS One. 2014 Oct 29;9(10):e110808. doi: 10.1371/journal.pone.0110808 (PMC4213012; doi:10.1371/journal.pone.0110808)

Figure S2. Coverage of chloroplast genomes. Reads from the "Tumor" experiment were mapped to a database of chloroplast genomes, and the coverage of genomes with more than 200 matches is depicted here. The *B. hypnoides* genome was removed from further analysis due to the observed uneven coverage.

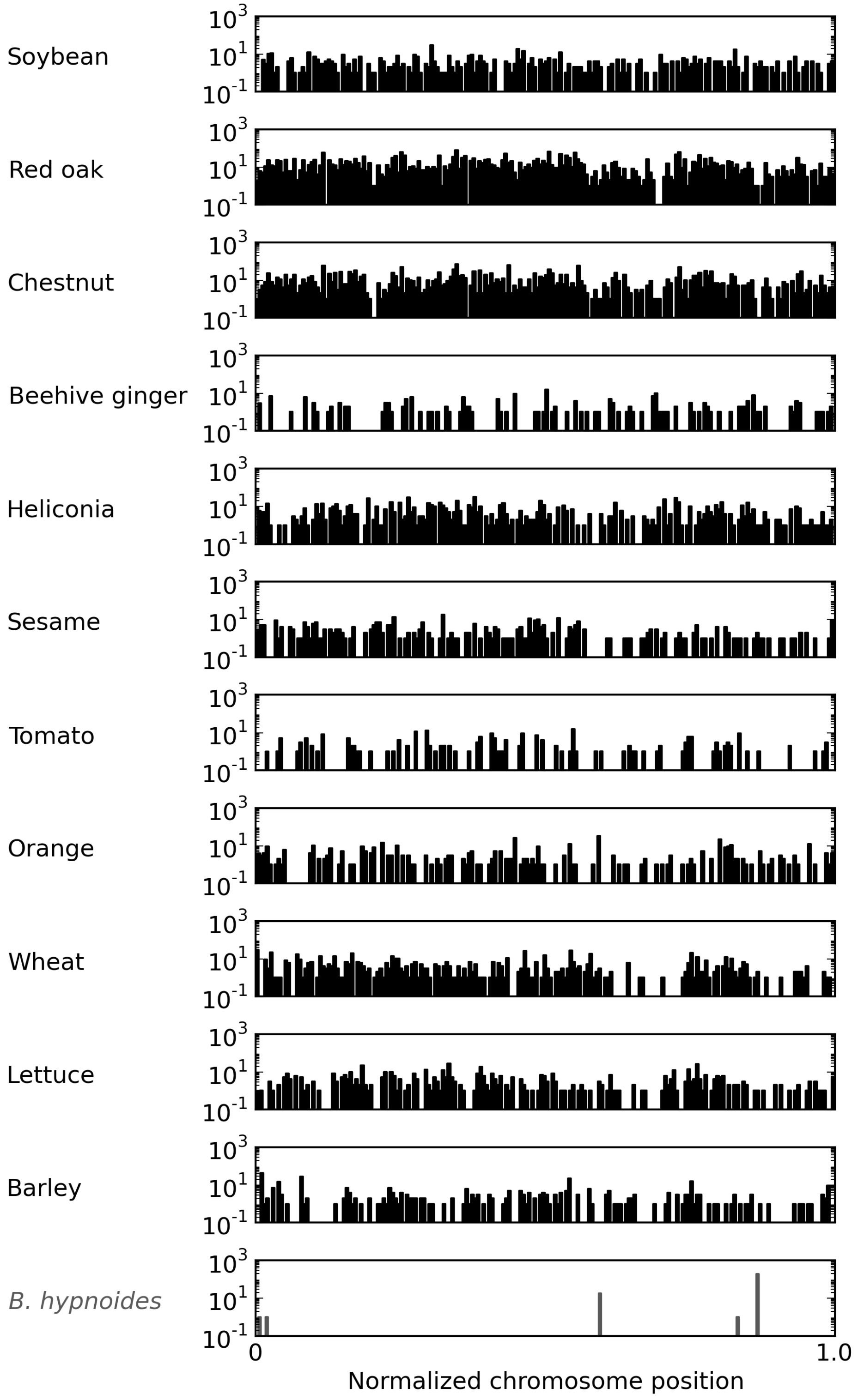

Supplement: Figure S2 — Coverage of chloroplast genomes. Reads from the “Tumor” experiment were mapped to a database of chloroplast genomes, and the coverage of genomes with more than 200 matches is depicted here. The B. hypnoides genome was removed from further analysis due to the observed uneven coverage. (PDF) [file pone.0110808.s002.pdf]
